# Brief Intervention to Prevent Sexually Transmitted Infections and Unintended Pregnancies: Protocol of a Mixed Methods Feasibility Study

**DOI:** 10.2196/15569

**Published:** 2020-03-10

**Authors:** Rob Stephenson, Nicholas Metheny, Tamar Goldenberg, Nataliia Bakunina, Sofia De Vasconcelos, Karel Blondeel, James Kiarie, Igor Toskin

**Affiliations:** 1 School of Nursing University of Michigan Ann Arbor, MI United States; 2 Center for Sexuality and Health Disparities University of Michigan Ann Arbor, MI United States; 3 MAP Centre for Urban Health Solutions St. Michael’s Hospital Toronto, ON Canada; 4 Carolina Population Center University of North Carolina at Chapel Hill Chapel Hill, NC United States; 5 UNDP-UNFPA-UNICEF-WHO-World Bank Special Programme of Research, Development and Research Training in Human Reproduction (HRP) World Health Organization Geneva Switzerland; 6 Higher School of Health Administration Institute of Leadership and Health Care Management First Moscow State Medical University (Sechenov University) Moscow Russian Federation; 7 Faculty of Medicine and Health Sciences Ghent University Ghent Belgium

**Keywords:** brief interventions, brief sexuality-related communication, sexual health, risky sexual behavior, STIs, unintended pregnancy

## Abstract

**Background:**

Sexual well-being is fundamental to physical and emotional health, and the ability to achieve it depends on access to comprehensive sexuality information and high-quality sexual health care from evidence-informed, nonjudgmental providers. Adequate and timely delivery of these components to individuals who are at high risk for sexually transmitted infections (STIs), including HIV, and unintended pregnancies promotes sexual health and mitigates consequences arising from risky sexual behavior. Brief interventions that allow health care providers to improve the information available to clients and motivate and help them to develop risk-reduction skills are seen as efficient ways to improve knowledge, change client behavior, and reduce provider stigma regarding sexual health.

**Objective:**

The aim of the study is to evaluate five aspects of feasibility (acceptability, willingness, safety, satisfaction, and process) of a brief sexuality-related communication (BSC) intervention based on motivational interviewing and behavior change techniques in primary health care settings in low- and middle-income countries (LMICs).

**Methods:**

This protocol outlines a multisite, multiphase study of feasibility of a BSC intervention in primary health care settings in LMICs that will be examined across four phases of the study. Phases I through III involve the collection of formative, qualitative data to examine provider and client perceptions of the feasibility of the intervention, adaptation of the intervention guide, and training providers on how to implement the final version of the BSC intervention. During phase IV, the feasibility of the intervention will be tested in a nonrandomized pre-post test trial where providers and clients will be followed for 6 months and participate in multiphase data collection.

**Results:**

Phase I is currently underway in Moldova, and phases I and II were completed in Peru in late 2019. Results are expected for the feasibility study in 2021.

**Conclusions:**

This feasibility study will determine whether the implementation of brief intervention programs aimed at improving sexual health outcomes is possible in the constraints of LMIC health systems and will add to our understanding of factors shaping clinical practice among primary care providers.

**International Registered Report Identifier (IRRID):**

DERR1-10.2196/15569

## Introduction

Sexual health is fundamental to the physical and emotional health of individuals, couples, and families and the social and economic development of communities and countries [1,2]. Sexual health encompasses the human rights of all persons to have the knowledge and opportunity to pursue a safe and pleasurable sexual life [2]. The ability to achieve sexual well-being depends on access to comprehensive sexuality information (knowledge about the risks and benefits and vulnerability to unintended consequences of sexual behavior) and high-quality sexual health care from evidence-informed, nonjudgmental providers [2-4]. The confluence of these components promotes sexual health and may avert two consequences that arise from risky sexual behavior: acquiring sexually transmitted infections (STIs), including HIV, and unintended pregnancies [1].

Brief interventions are seen as efficient ways to improve knowledge, change behavior [5-12], and reduce provider stigma regarding sexual health [13,14]. When built on evidence-based behavioral change techniques [15] and delivered using brief sexuality-related communication (BSC) [3] tools, brief interventions address client-driven sexual health goals in a single session (less than 25 minutes) between clients and their health care provider. Brief interventions often use techniques based on motivational interviewing (MI). This client-centered approach enhances intrinsic motivation to change by exploring and resolving ambivalence and allows health care providers to improve the information available to clients, motivate clients, and help clients develop the concrete skills necessary to change risk behaviors [5,8,11].

However, much of the evidence on brief interventions comes from countries with highly developed health care systems [11,16]. There is little known about how brief interventions can be tailored to improve sexual health in LMICs. There is also little literature on how providers in LMICs view their ability to embed such an intervention in their practice settings and health system environments. Characterized by competing demands for limited resources and a shortage of many types of primary health care providers [17-19], the health systems of many LMICs may be initially ill-equipped to widely implement the proposed intervention, thus requiring important alterations to any brief intervention. Likewise, the degree to which a brief intervention should be tailored to populations with specific sexual health needs (eg, sex workers, men who have sex with men [MSM]) versus standardized across populations to ease implementation and reduce provider burden is unknown in these settings.

To address the limitations of the literature, we propose a multisite, multiphase feasibility study of a brief intervention designed to improve sexual health in various settings. The feasibility study is grounded in the information-motivation- behavioral skills (IMB) model [20]. This model borrows from established behavior change theories [20] such as the theory of reasoned action [21] and the concept of self-efficacy [22] and has received considerable attention in HIV prevention and antiretroviral medication adherence [23-31].

Information is a necessary but insufficient precursor to preventive behavior and risk reduction [20,30]. It can include specific facts about the consequences of the risk behavior in question that serve as a guide for personal preventive actions and cognitive processes that significantly influence performance of preventive behavior [27]. Just as important as factual information, however, is misinformation regarding the risk behavior in question [23]. Inaccurate information regarding prevention of and care for STIs, HIV, and unintended pregnancies can negatively influence this component of the IMB model, reducing the chances of a successful change in preventive behavior [23,32].

Motivation is a function of two components that influence incentive to practice preventive behaviors: behavioral intention and subjective norms [21]. These components reflect the degree to which personal motivation (ie, feelings about the preventive action in the context of competing life demands) and social motivation (ie, social support, level of stigma associated with the preventive action, or the consequences of inaction) facilitate the adoption of a preventive behavior [23]. The development of behavioral skills is an additional step that aids in behavior change. It involves the objective ability and perceived self-efficacy concerning performance of the behavior [20]. The ability to obtain behavioral skills is not limited to individuals themselves (ie, knowledge of how to effectively use condoms) but can involve structural elements as well (ie, access to condoms or social norms that discourage condom use in marriage) [33].

The IMB model stipulates that information and motivation work primarily through behavioral skills to influence behavior. Associations between information and motivation theoretically lead directly to behavior change, although these pathways are less robust in structural equation modeling analysis than the indirect pathways via behavioral skills [20].

The BSC intervention itself is based on techniques of MI. Developed by Miller and Rollnick [34], MI is defined as a “directive, client-centered counseling style for eliciting behavior change by helping clients to explore and resolve ambivalence” [35,36] and aims to increase clients’ intrinsic motivation to stimulate change from within rather than being imposed externally [35]. In a single 20- to 25-minute session, providers will move through a series of steps designed to elicit clients’ life goals and current sexual risk behaviors. These will be used to fashion an individualized concrete action plan to reduce risk behaviors in service to their larger life goals. In addition to reducing client sexual risk behavior, the intervention aims to change how providers communicate with clients regarding their sexual health in an empathic, respectful, and nonjudgmental manner. By altering client-provider communication to be less didactic and more individualized, providers may increase clients’ intrinsic motivation for behavior change.

The aim of the study is to evaluate 5 aspects of feasibility (acceptability, willingness, safety, satisfaction, and process) of a BSC intervention based on MI and behavior change techniques in primary health care settings in LMICs.

This document is a generalized protocol, applicable to all potential study sites. Study locations will be chosen at a later date in consultation with in-country researchers and providers. This will lead to the creation of country-specific protocols developed over a series of 2-day site meetings with officials from the national, regional, and local Ministries of Health to clearly delineate roles, responsibilities, and alterations to the general protocol that may be necessary in each selected study community. This paper outlines the master protocol: the overall research approach that will be used to determine the feasibility of the BSC. The protocol will be adapted accordingly for individual countries.

## Methods

### Testing the Feasibility of a Brief Sexuality-Related Communication Intervention

The purpose of this study is to test the feasibility of a BSC intervention. Feasibility in this study will be guided by 5 technical principles: acceptability, willingness, safety, satisfaction, and process (study logistics). These aspects of feasibility will be examined across all phases of the study. This study was approved by the Ethics Review Committee (ERC) of the World Health Organization (WHO).

The feasibility study will follow a 4-phase, iterative design. These phases are structured to refine the intervention for optimal implementation with providers and clients in primary care settings. Phases I through III will involve the collection of formative, qualitative data to examine provider and client perceptions of the feasibility of the intervention and their attitudes toward sexual health and perceived sexual health information needs. Data will be collected using an iterative approach, with data from the previous phase being analyzed and the study refined before beginning the subsequent phase. After phase III, providers will be trained on how to implement the final version of the BSC intervention. After the final phase of formative, qualitative work (phase III), the feasibility of the intervention will be tested in a nonrandomized pretest-posttest trial of the BSC intervention (phase IV).

### Study Sites

The feasibility study will be implemented across multiple countries and multiple study communities within each country. For the purposes of this protocol, the term study site refers to an individual primary health care center. Study populations in each study country will be selected according to national sexual health priorities within the bounds of the proposed client population and sexual health outcomes. All research, training, and intervention documents will be translated from English to the appropriate local languages by local research teams and then backtranslated to English and compared for accuracy.

### Participants

Participants will include key informants in phase I and health care providers and clients across all 4 study phases. Key informants include stakeholders at local, regional, and national levels. Stakeholders will include decision makers and experts at health facilities and clinics, health departments, and other sexual and reproductive health organizations. In accordance with WHO guidelines on BSC [3], participating health care professionals will include those at the first point of client contact. This definition includes physicians, nurses, nurse practitioners, and other providers (eg, HIV testing counselors, family planning counselors) who offer primary health care. The aim of the intervention is to improve sexual health by reducing the burden of STIs and unwanted pregnancies. Client populations will vary by country but will include specific populations in each country who experience a high burden of STIs and/or HIV or want to prevent pregnancy. For example, key client populations experiencing high levels of STI/HIV may include sex workers, MSM, and people seeking treatment for STIs. Clients experiencing high levels of unwanted pregnancy will be women (including adolescent women) seeking family planning services (who may also be targeted for STI prevention). The intervention is standardized to populations of interest for each country based on conversations with key stakeholders, with minor differences in language and content depending on the specific client population and country. These differences in language and content are designed to promote cultural sensitivity, address unique issues of stigma in each location, and tailor the intervention to the specific sexual health issues facing each population.

### Recruitment

Key informants will be recruited using existing contacts of the in-country key stakeholders and study staff who, in turn, will reach out to possible key informants on local, regional, and national levels to invite them to share their expertise. Clients and providers will be recruited at each phase of the feasibility study. That is, while clients and providers will not be prevented from participating in more than one phase, additional clients and providers will be recruited at each stage of the feasibility study. Recruitment of providers and clients will occur through primary health care sites and organizations. To avoid the potential coercion that may occur with the unequal power balance between provider and client, providers will not recruit their own clients into the study.

Recruitment sites may include those that address the specific needs of the selected study populations, such as general primary care clinics, health care clinics, organizations addressing issues of STIs and unintended pregnancy, pharmacies, and mobile outreach clinics. Recruitment of providers and clients in these sites will occur with the assistance of a key stakeholder at each site; recruitment of clients will also be facilitated by study staff. Potential provider participants will be approached by study staff and brought to a room at the health care site with audio and video privacy to explain the study, establish eligibility, and provide informed consent if the participant agrees to participate.

For recruitment of clients, the key stakeholder will assist by identifying appropriate times to approach potential client participants with study recruitment materials in the waiting areas of the primary health care sites. Study staff will be stationed in these waiting areas and, using a predetermined protocol, will approach every fourth person who enters the waiting area. Study staff will take the potential participant to a space in the same building with audio and visual privacy to screen the participant for eligibility, explain the study, and provide informed consent should the potential participant agree join the study.

All participants (including providers and client populations) will receive locally appropriate reimbursements for their time and effort participating in research activities; reimbursements are not meant to be a motivating factor for enrollment in the study.

### Sample Size

Sample sizes are approximate and will differ by study country, study community, and study site. As the goals of this study are to inform the feasibility, willingness, and acceptability of the BSC protocol among providers and clients, sampling is not intended to maximize external validity or provide data that are generalizable. However, it is important that the feasibility data are reflective of a diverse range of opinions and lived experiences. In sampling for the feasibility study, a diverse sample will be sought in terms of age and locally specific race/ethnicity in order to gather a range of opinions regarding both the formative qualitative (phases I to III) and quantitative (phase IV) portions.

### Procedures

The study will be jointly implemented by an international team of researchers, key stakeholders in each study country already known to the international research team, and in-country study staff who have specific knowledge of the study populations and health care system in each study community. This implementation strategy will occur across all phases of the study.

### Phase I

During the first phase of the study, 3 study activities will take place: key informant interviews with stakeholders in each study community, individual in-depth interviews with health care providers, and focus group discussions with client populations.

Key informant interviews are in-depth, qualitative interviews used to better understand the contexts in which the intervention will be implemented: to address the appropriateness and safety of the intervention in facility settings, perceived usefulness of the intervention, special considerations for adaptations to study communities, and barriers and facilitators for implementing the intervention. Key informant interviews will be conducted by the national research team with approximately 10 local, regional, and federal stakeholders in each community for about 30 minutes. Interviewers will use a semistructured interview guide that will be identical across study sites but adapted based on the stakeholder level (local, regional, federal).

Individual in-depth interviews will address provider perceptions around participating in the research study, participating in the intervention training, and providing the intervention. In-depth interviews will assess attitudes toward providing sexual health information to clients and providers’ perceived sexual health needs of their clients and their comfort in working with vulnerable populations (eg, MSM). In-depth interviews will be stratified by study site and provider type (doctor, nurse, and lay counselor), with approximately 15 in-depth interviews for each study site (5 per group). Interviews will be conducted using semistructured interview guides, occur in locations with audio and visual privacy, and last approximately 45 to 60 minutes. Addressing all key domains of feasibility (acceptability, willingness, safety, satisfaction, and process), the interviews will examine: (1) overall attitudes about the intervention, (2) willingness and motivation to participate in the research study and implement the intervention, (3) logistics of implementation, (4) ability to implement the intervention, and (5) attitudes about providing sexual health services.

Focus group discussions with clients will be used to increase understanding of clients’ general perceptions about the intervention. Three focus group discussions will be conducted in each study community with approximately 24 to 30 clients seeking to alleviate concerns regarding stigmatization and discrimination that may occur in some study communities (eg, MSM, sex workers) and explore similarities in sexual risk behavior and sexual health needs. Focus group discussions lasting approximately 60 to 90 minutes will be conducted by trained moderators (one from each of the local research teams) using a semistructured focus group discussion guide. Guides will be the same across study communities and translated into the local language. During focus group discussions, the moderator will describe the process of the BSC intervention and illustrate how the intervention works by conducting a short role play with an example of the intervention. Participants will then be asked about their reactions to the process and logistics of the research study, the intervention, and the general content of the intervention. Participants will be asked to rate specific aspects of the intervention based on its perceived importance and usefulness, comfort with the intervention, perceived safety of the intervention, and willingness to participate in the intervention. Participants will also discuss their perceived sexual health information needs and their comfort in talking about sexual health with their providers.

### Phase II

Phase II uses cognitive interviewing, in which feedback is sought regarding the language, phrasing, and delivery of the BSC guide to each of the study populations [37]. The aim of the cognitive interviews is not to generate substantive data on attitudes or perceptions but to test comprehension of language used in the intervention. Cognitive interviews will be conducted among providers (n≈4) and clients (n≈8) in each study community for approximately 30 minutes. The number of proposed cognitive interviews is a suggestion and may be adapted for each country depending on the degree of heterogeneity identified in comprehension of the intervention language. The number of cognitive interviews may also need to be expanded for countries with multiple languages. Cognitive interviews differ from traditional interviews in that key passages of the BSC guide will be read to the interviewee with the intent of ensuring the message of the passage comes across as intended. The goal of these interviews is not to collect data from participant responses but rather information on how they respond and why. This will help to elicit information on the validity of the language in the guide as translated and improve cultural sensitivity of the intervention by including site-specific terminology. To avoid repetitiveness and conditioning a social desirability bias [38,39] and to mitigate task burden on the part of interviewers, only selected modules from the BSC guide will be reviewed during the cognitive interview. These will be selected in advance by the research team to give a broad view of the types of words, phrases, and language used in the intervention.

### Phase III

Phase III consists of theater testing commonly used to gauge participant responses to an intervention session [40] and will be performed among providers and clients for approximately 60 to 90 minutes. The aim of this phase is to obtain an assessment of willingness and acceptability of participants to take part in the intervention. During theater testing, in-country study staff will demonstrate a mock BSC session (via a video recorded BSC session). The participants, who will be selected to represent potential study participants, will provide feedback on the content, delivery, and materials used in the BSC intervention and engage in group dialogue surrounding potential improvements to the guide.

Four to six providers per study community will participate in theater testing. For clients, groups will be stratified based on the population of interest. There will be three separate groups per study community (n≈8-10 participants per group), with one group for each study population. During these client theater testing sessions, the standardized client persona in the mock BSC session will be matched to the population of that particular focus group.

### Intervention Training

After revisions to the intervention based on phases I to III are complete, training will take place at each study site with recruited providers. The training package consists of 12 modules delivered over three days, covering the burden of STIs in the local context, the WHO sexual health framework [2] and BSC principles [3], importance of creating nonjudgmental clinical spaces, client confidentiality, ways to reduce implicit and explicit biases in delivering sexual health care, and finally, how to conduct the BSC intervention. Training will be a combination of didactic learning and role playing: trainees will have the opportunity to role play through a number of scenarios (eg, different sexual health needs and/or different client groups [eg, MSM]).

### Phase IV: Testing the Intervention

Phase IV will test the feasibility of the intervention. During phase IV, trained providers will deliver the intervention as revised in phases I to III as part of regularly scheduled consultations with clients in one of the study populations. To test the feasibility of the intervention during this phase, providers and clients will be followed for 6 months and participate in a variety of study activities. Providers will participate in knowledge, attitude, and practice (KAP) surveys, and a subset will participate in intervention observations. Intervention observations are intended to assess the fidelity to the intervention. Clients will participate in pre-post surveys and exit interviews upon completion of an intervention session. Separate measures are used to elicit data on the intervention from providers and clients (eg, willingness to participate in the intervention).

### Knowledge, Attitude, and Practice Surveys for Providers

Prior to beginning training, providers will complete KAP surveys that will serve as the pretest for the intervention. Follow-up KAP surveys will be administered to providers at 3 and 6 months posttraining in order to examine changes in the measures of interest. A 6-month follow-up period was chosen to facilitate measurement of short-term gains in MI techniques and identify changes in attitudes and perceptions about providing sexual health services to client populations. The surveys will cover 6 domains of the provider experience with the intervention, provider-client interaction, and the providers’ perceived utility of the intervention to both the provider and the client, including sociodemographic information, provider skills, efficacy and autonomy, competency and capacity, implementation, and attitudes. Provider intervention measures include indicators such as willingness to deliver the intervention and comfort with MI techniques (willingness), perceptions of the intervention’s impact on client behavior (satisfaction), perceptions of the intervention’s fit with the facility’s culture and clinical requirements (acceptability), and ability for the study site to continue delivering the intervention after the research study concludes (intervention logistics).

### Intervention Observations

During phase IV, in-country study staff will observe 10% of BSC sessions to independently assess provider knowledge, practice, and fidelity to the BSC protocol as written. The goal of the intervention observations is to assess the degree to which providers retain fidelity to the study protocol and provide nonjudgmental care consistent with the technical principles of MI. In-country study staff will use a standardized checklist to determine fidelity to the BSC guide. The checklist will include all the behavior skills taught in the training and the expected steps of the BSC session. During the observations, staff will be positioned so as not to interfere with the client-provider interaction, and the client will be asked if they consent to having the study staff member present. Intervention observations will collect quantitative data regarding provider ability to deliver the intervention protocol as written. Staff will check off skills as they are observed and record whether the steps of the BSC intervention are followed and are followed in order. At least two intervention observations per client type will be performed at each study site. Any issues regarding cultural sensitivity for key populations will be noted by study staff during the intervention observations. Providers who consistently demonstrate nonadherence to the intervention will be offered a 1-day refresher training for the intervention.

### Pre-Post Surveys With Clients

A convenience sample of clients from each study community will be recruited, and pre-post surveys will be administered prior to participation in the intervention (at baseline) and at 3 and 6 months postintervention. Adaptive, computer-assisted self-interviewing software with audioenabled playback will be used to address language barriers and issues of client literacy. The survey will take approximately 20 minutes to complete and include questions that assess (1) client sociodemographics and willingness, satisfaction, safety, and acceptability of the intervention (domains 1 to 5) and (2) client sexual health, including sexual competency and sexual behaviors (domain 6). Approximately 90% of questions will be the same across the key populations of clients; however, there will be some differences in questions regarding sexual behaviors depending on whether the outcome of interest is prevention of STIs, unintended pregnancy, or both.

### Exit Interviews

Exit interviews will qualitatively assess client reactions to the intervention and the research study. Interviews will be completed upon receiving the intervention in order for clients to discuss their immediate feelings and attitudes toward the intervention. Approximately 24 interviews lasting 45 to 60 minutes will be conducted per study community, approximately 6 per key client population, by a member of the local research team. Interviewers will use an identical semistructured interview guide translated into the local language. Exit interviews will address client satisfaction with and willingness to use the intervention and client perception of intervention acceptability and safety.

### Study Documentation

Data will be collected regarding the recruitment and retention of clients and providers. These data will be collected by in-country study staff at each site using standardized checklists that track the number of clients and providers approached, number of those who agree to participate in the study, number who refuse, and the main reason for refusal. Participant contact information will be collected in order to provide reminders for the follow-up surveys. During the follow-up period, client and provider subject identification numbers will remain consistent in order to track retention through the 6-month follow-up period. In case of attrition, an attempt will be made to collect data on the reasons for discontinuing the study.

### Ethics and Consent to Participate

The core BSC protocol was first submitted to the WHO Research Project Review Panel; after its technical approval in 2016, WHO ERC was consulted for a special evaluation of the ethical components, with the following approval in 2017: prior to participation in any study activities, all participants (providers, clients, and key informants) will provide written informed consent at all stages (phases I to IV) of the study. The informed consent forms are explained in detail, and participants are asked to read them in full before agreeing to sign. If the individual chooses to participate, they sign the informed consent form and are offered a copy of the signed form. They can choose to decline taking a copy of the form if there is any concern that this would create additional risks.

## Results

Phase I is currently underway in Moldova, and phases I and II were completed in Peru in late 2019. Results are expected for the feasibility study in 2021.

### Analysis of Phases I and III

All focus group discussions and in-depth interviews will be audiorecorded, transcribed verbatim, and deidentified. For the focus group discussions, a notetaker will be present and will indicate the order of speakers and the first few words that each speaker says in order for the transcriber to be able to differentiate between speakers. For all activities in phases I and III with the exception of cognitive interviewing, a thematic analysis of these transcripts will be completed, using elements of grounded theory [41] and building on the IMB model. This will include the systematic and consistent application of deductive and inductive codes to the text. Inductive codes will include themes that are explicit domains present in the interview and focus group discussion guides, and deductive codes will include salient themes that arise more organically in the data. Additionally, all inductive themes and codes will be grouped according to the constructs in the IMB model, including information, motivation, and behavioral skills.

A preliminary codebook will be developed with provisional definitions for each code. A team of 2 to 5 data analysts will apply the provisional codebook to a single transcript, and the coded transcripts will be merged for comparison. Analysts will examine and discuss discrepancies in coding, and the code definitions will be revised based on an examination of coding disagreement. The process will be repeated with the revised codebook until consistent agreement among coders is attained. This process will occur with transcripts from all data collection activities using the same codebook for transcripts across all data collection activities. Once the final codebook is established, codes will be applied to all transcripts, with at least 2 analysts coding each transcript.

Based on systematic close readings of coded text, analysts will create thick descriptions for each theme. These descriptions will identify common concepts, patterns, and unique statements that appear in the transcripts. Specific themes arising in phases I to III regarding content and delivery of the intervention will be used by the research team to further refine the intervention for phase IV, determine the best way to present content, and better understand provider/client attitudes and willingness to participate. While small changes will be site-specific in order to promote cultural sensitivity, larger changes will be implemented universally across study sites.

### Analysis of Phase II

Analysis of phase II will be completed separately from the in-depth interviews and focus group discussions. For each interview, interviewer notes will be synthesized and grouped into themes based on the content of the participant feedback. These themes will be used for making recommendations to improve the intervention. Specifically, these recommendations will involve possible changes to the phrasing and language of the intervention, with the goal of improving comprehension and acceptability of the intervention among providers and clients. While small changes in the language, phrasing, and delivery of the intervention will be site-specific in order to promote cultural sensitivity, larger changes will be implemented universally across study sites to maintain protocol fidelity.

### Analysis of Quantitative Data From the Pre-Post Surveys and Exit Interviews

Data (client and KAP surveys and client exit interviews) will be deidentified and entered into the study database at each time point (0, 3, and 6 months). At each time point, descriptive statistics will be computed, and 3- and 6-month follow-up data will be compared with previous survey data. Appropriate tests of comparison (ie, *t* tests, chi-square tests, analyses of variance) will be used to determine differences in the primary (feasibility) and secondary (sexual health) outcome measures between time points. Although this phase is not powered to detect statistically significant differences in the sexual health outcomes, this analysis will provide information on whether the intervention is associated with differences in these outcomes from baseline to 3- and 6-month follow-up. Once collected by in-country study staff, the quantitative data from the intervention observations will be deidentified and entered into the study database.

## Discussion

### Feasibility Study Implications

Brief interventions provide an opportunity to train providers on the topic of sexual health for different populations, including marginalized populations for which they may have received little to no training. By training providers to improve their communication with these populations, providers may be able to work with clients to create plans of action that reduce risk behaviors in service to the clients’ larger life goals. However, the scale and novelty of this project in LMICs requires a feasibility study to determine if the implementation of wider brief intervention programs is possible given the constraints of LMIC health systems and the demands already placed on primary care providers. This protocol, therefore, lays out an innovative approach to refining existing methods for implementing brief interventions for use in more resource-constrained settings. By taking an iterative approach, this feasibility study allows for the alteration of the protocol to best fit the needs of the target populations, both client and provider.

### Limitations

Despite these strengths, the feasibility study does have limitations. Reliance on existing health care resources in the study communities may show that there is a demand among clients but a lack of feasibility among health systems and providers. This would require a different approach altogether—one outside of the health system. The ability to recruit and retain providers and participants may also be challenging given the multiple follow-up time points. The study only follows clients and providers for 6 months: this shorter follow-up period is expected to increase retention but will preclude the ability to identify longer term changes in provider’s skills and attitudes. While this remains a potential limitation, the research team will work closely with the in-country study team to fashion reimbursements and contact methods to maximize retention throughout the study period.

### Conclusions

Using brief interventions to reduce provider stigma and sexual risk behaviors among marginalized populations has the potential to be a cost-effective approach to improve sexual health in resource-constrained settings. The collection of formative, qualitative data that directly inform the content and delivery of the brief intervention may increase our understanding of how brief interventions can be delivered by health care providers in resource-limited health systems and different sociocultural contexts. Testing of the feasibility of the BSC in multiple settings and across multiple population groups will provide vital information on best practices for implementing brief interventions that are culturally sensitive and meet the needs of a range of vulnerable groups (eg, MSM or adolescent women). The data will also highlight factors shaping clinical practice among primary care providers and allow for the creation of more concrete, effective action plans designed to reduce sexual risk behaviors.

This feasibility study will determine whether the implementation of brief intervention programs aimed at improving sexual health outcomes is possible in the constraints of LMIC health systems. Understanding the ability of primary health care providers to deliver brief interventions and factors shaping clinical practice that will be investigated in this study is an important step in the improving the quality of sexual health services in resource-limited settings. While both provider and client perceptions of the feasibility of the intervention will be examined, adaptation of the intervention guide based on the feedback of the study populations and training providers on how to implement the final version of the BSC intervention will be performed during the study. However, careful attention will also be needed to the sustainability of the intervention. The current protocol will establish whether the integration of a BSC into routine patient visits is feasible. If proven feasible, strategies will need to be in place for sustainability, including routine trainings, training refreshers for providers, and close collaboration with key stakeholders (eg, ministries of health). Brief interventions will allow health care providers to improve the information available to clients, motivate and help them to develop risk-reduction skills that are seen as efficient ways to improve knowledge, change clients’ behavior, and reduce provider stigma regarding sexual health.

## Appendix

Multimedia Appendix 1 Approval of the study master-protocol by the Research Proposal Review Panel (RP2) of the World Health Organization’s Department of Reproductive Health and Research (RHR; included two independent reviewers). RP2 approval is a condition sin qua non to continue a project within RHR and RP2 was also responsible for approving the proposed budget for the study.

Multimedia Appendix 2 RP2 approval of the site-specific Protocol for Peru.

Multimedia Appendix 3 Approval of the core protocol by World Health Organization Research Ethics Review Committee.

## Abbreviations

BSC: brief sexuality-related communication
ERC: Ethics Review Committee
IMB: information-motivation-behavioral skills
KAP: knowledge, attitude, and practice
LMIC: low- and middle-income country
MI: motivational interviewing
MSM: men who have sex with men
STI: sexually transmitted infection
WHO: World Health Organization

## References

[1] (2010). World Health Organization.

[2] (2017). World Health Organization.

[3] (2015). World Health Organization.

[4] Toskin I, Cooper B, Troussier T, Klugman B, Kulier R, Chandra-Mouli V, Temmerman M (2015). WHO guideline for brief sexuality-related communication: implications for STI/HIV policy and practice. Reprod Health Matters.

[5] Picciano J, Roffman R, Kalichman S, Rutledge S, Berghuis J (2001). A telephone based brief intervention using motivational enhancement to facilitate HIV risk reduction among MSM: a pilot study. AIDS and Behavior.

[6] Harvey D, Tsey K, Cadet-James Y, Minniecon D, Ivers R, McCalman J, Lloyd J, Young D (2002). An evaluation of tobacco brief intervention training in three indigenous health care settings in north Queensland. Aust N Z J Public Health.

[7] Deen D, Lu W, Rothstein D, Santana L, Gold MR (2011). Asking questions: the effect of a brief intervention in community health centers on patient activation. Patient Educ Couns.

[8] Monti PM, Colby SM, Barnett NP, Spirito A, Rohsenow DJ, Myers M, Woolard R, Lewander W (1999). Brief intervention for harm reduction with alcohol-positive older adolescents in a hospital emergency department. J Consult Clin Psychol.

[9] Henry-Edwards S, Humeniuk AR, Monteiro M, Poznya V (2003). World Health Organization.

[10] Lightfoot M, Rotheram-Borus MJ, Comulada WS, Reddy VS, Duan N (2010). Efficacy of brief interventions in clinical care settings for persons living with HIV. J Acquir Immune Defic Syndr.

[11] Vasilaki EI, Hosier SG, Cox WM (2006). The efficacy of motivational interviewing as a brief intervention for excessive drinking: a meta-analytic review. Alcohol Alcohol.

[12] Dunn C, Deroo L, Rivara FP (2001). The use of brief interventions adapted from motivational interviewing across behavioral domains: a systematic review. Addiction.

[13] Poustchi Y, Saks NS, Piasecki AK, Hahn KA, Ferrante JM (2013). Brief intervention effective in reducing weight bias in medical students. Fam Med.

[14] Shah SM, Heylen E, Srinivasan K, Perumpil S, Ekstrand ML (2014). Reducing HIV stigma among nursing students: a brief intervention. West J Nurs Res.

[15] Michie S, Richardson M, Johnston M, Abraham C, Francis J, Hardeman W, Eccles MP, Cane J, Wood CE (2013). The behavior change technique taxonomy (v1) of 93 hierarchically clustered techniques: building an international consensus for the reporting of behavior change interventions. Ann Behav Med.

[16] (2004). Government of Australia Department of Health.

[17] (2009). United Nations Population Fund.

[18] (2019). United Nations Development Programme.

[19] (2016). United Nations Department of Economic and Social Affairs.

[20] Fisher JD, Fisher WA (1992). Changing AIDS-risk behavior. Psychol Bull.

[21] Ajzen I, Fishbein M (1980). Understanding Attitudes and Predicting Social Behaviour.

[22] Bandura A (1977). Self-efficacy: toward a unifying theory of behavioral change. Psychol Rev.

[23] Rivet Amico K (2011). A situated-information motivation behavioral skills model of care initiation and maintenance (sIMB-CIM): an IMB model based approach to understanding and intervening in engagement in care for chronic medical conditions. J Health Psychol.

[24] Ballester-Arnal R, Gil-Llario MD, Salmeron-Sánchez P, Giménez-García C (2014). HIV prevention interventions for young male commercial sex workers. Curr HIV/AIDS Rep.

[25] Fisher JD, Fisher WA, Misovich SJ, Kimble DL, Malloy TE (1996). Changing AIDS risk behavior: effects of an intervention emphasizing AIDS risk reduction information, motivation, and behavioral skills in a college student population. Health Psychol.

[26] Fisher W, Williams S, Fisher J, Malloy T (1999). Understanding AIDS risk behavior among sexually active urban adolescents: an empirical test of the information-motivation-behavioral skills model. AIDS and Behavior.

[27] Fisher W, Fisher J, Harman J (2003). The information-motivation-behavioral skills model: a general social psychological approach to understanding and promoting health behavior. Soci Psychol Foundations Health Illness.

[28] Fisher JD, Fisher WA, Amico KR, Harman JJ (2006). An information-motivation-behavioral skills model of adherence to antiretroviral therapy. Health Psychol.

[29] Gil-Llario MD, Ballester-Arnal R, Giménez-García C, Salmerón-Sánchez P (2014). Effectiveness of HIV prevention for women: what is working?. AIDS Behav.

[30] Kalichman S, Malow R, Dévieux J, Stein JA, Piedman F (2005). HIV risk reduction for substance using seriously mentally ill adults: test of the information-motivation-behavior skills (IMB) model. Community Ment Health J.

[31] Nöstlinger C, Nideröst S, Gredig D, Platteau T, Gordillo V, Roulin C, Rickenbach M, Dias SF, Rojas D, Swiss HIV Cohort Study, Eurosupport5 Study Group (2010). Condom use with steady partners among heterosexual people living with HIV in Europe: testing the information-motivation-behavioral skills model. AIDS Patient Care STDS.

[32] McMahan V, Goicochea P, Vargas L, Marcus JL, Grant RM, Liu A (2012). Supporting study product use and accuracy in self-report in the iPrEx study: next step counseling and neutral assessment. AIDS Behav.

[33] Fisher J, Fisher W, Peterson JL, DiClemente RJ (2000). Theoretical approaches to individual-level change in HIV risk behavior. Handbook of HIV Prevention.

[34] Bennett G, Miller WR, Rollnick S (1991). Motivational Interviewing: Preparing People to Change Addictive Behavior.

[35] Miller W, Rollnick S (2020). Motivational Interviewing: Preparing People For Change, 2nd Edition.

[36] Berg RC, Ross MW, Tikkanen R (2011). The effectiveness of MI4MSM: how useful is motivational interviewing as an HIV risk prevention program for men who have sex with men? A systematic review. AIDS Educ Prev.

[37] Beatty PC, Willis GB (2007). Research synthesis: the practice of cognitive interviewing. Public Opin Q.

[38] Campanelli P (2016). Testing survey questions: new directions in cognitive interviewing. Bull Sociol Methodol.

[39] Sudman S (2020). Thinking About Answers: The Application Of Cognitive Processes To Survey Methodology.

[40] Wingood GM, DiClemente RJ (2008). The ADAPT-ITT model: a novel method of adapting evidence-based HIV interventions. J Acquir Immune Defic Syndr.

[41] Charmaz K (2014). Constructing Grounded Theory.

