# Supplementary material for: Brief Intervention to Prevent Sexually Transmitted Infections and Unintended Pregnancies: Protocol of a Mixed Methods Feasibility Study
Source: JMIR Res Protoc. 2020 Mar 10;9(3):e15569. doi: 10.2196/15569 (PMC7093772; doi:10.2196/15569)
Supplement: Multimedia Appendix 2 [file resprot_v9i3e15569_app2.pdf]

FOR WHO USE ONLY
Date received by WHO:
Thematic area:
☐ Single site proposal
☐ "Core" proposal (for multicentre study)
☒ Centre-specific proposal under multicentre study

1. Connect ID No: A-ID: A65925.1.PE1

1. **Project title:** The BSC study

Feasibility Study of a Brief Intervention to Prevent STIs and Unintended Pregnancies in Peru

This review concerns the implementation protocol for the site in Peru

2. Principal investigator: Carlos Caceres

3. REVIEWER NAME: Elisabeth Faxelid

REVIEWER NAME: Anna Glasier

The implementation protocol for Peru has been approved on the 30th of November, 2017

Dr Anna Ekéus Thorson MD, MPH, PhD, Prof **I Research Manager I Research Capacity Strengthening I HRP Alliance I** Department of Reproductive Health and Research I Office: +41227911393 Cell: +41792173401

REVIEWER NAME: Elisabeth Faxelid

REQUESTED BUDGET:

PROJECT DURATION (IN MONTHS):

DATE OF REVIEW: 2017-11-28

INSTRUCTIONS: *Please review the proposals focusing on major strengths and weaknesses. Comments should be typed in the spaces below each section. Type as much as needed, the document will allow for unlimited text.*

PROJECT SUMMARY: *Please provide a brief statement of the main aim and objectives. Is there a good review of the literature, including reference to a systematic review if applicable, justifying the research questions?*

The overall aim is to evaluate the feasibility of a Brief Sexuality-Related Communication (BSC) intervention to be implemented by providers working in specified clinics of public facilities in Peru. This is done through a 4-phased study including providers as well as clients with elevated risk for STIs and/or unintended pregnancies. The 3 first phases has qualitative approach while the 4th phase is a pre- and post test pilot intervention.

Review of literature and references are relevant justifying the research question.

PROJECT SIGNIFICANCE: *Is the significance of the project accurately described?* ***Yes*** *Does it include links to research agendas of relevance to SRHR****?*** **Yes** *Will it provide new knowledge?* ***Yes***

OVERALL PROJECT DESIGN: *Is the proposal scientifically sound and is the design being proposed the most appropriate to accomplish the stated objectives****?*** **Yes, except that I have doubt about what conclusions can be drawn from the pilot intervention regarding sexual outcome since sample size is low and sampling method is hardly quantitative.** *Please provide a critique of the conceptual approach and the proposed project structure in relation to the objectives the project seeks to achieve. Have checklists (STROBE, PRISMA, etc) accurately informed the methods description?*

This is a feasibility study. It is a series of qualitative data collection followed by a small PILOT test – the intended outcomes are an understanding of what the feasibility of the intervention is: in terms of acceptability, willingness, safety and logistics. This information will be used to inform the development of a larger, efficacy powered RCT.

SAMPLING PROCEDURES: *Is the sampling, (including representativeness, if required) and the sample size, adequately described and justified?* **See comments above.** *Are adequate procedures outlined to recruit the respondents?* ***Yes*** *If a qualitative study, is selection of participants and method for gathering data accurately described?* **Yes**

DATA COLLECTION: *Do you foresee any difficulties in the proposed field activities, especially in the plans for data collection?* **No, but I think the researchers need a couple of more months in the end to finish the analysis and write up the report.**

ANALYSES, MAIN VARIABLES or OUTCOMES: *Is information provided on methods of analyses?* ***Yes*** *For quantitative studies, intended statistical methods?* ***Yes*** *Are the main variables or outcomes properly described?* ***Yes***

For qualitative studies, are scientifically sound methods of data collection analyses described, aiming for robustness and trustworthiness. ***Yes***

RESEARCH STANDARDS: *Do you see any ethical challenges in the project description?* ***The populations included are vulnerable and stigmatized but the authors have taken this aspect into account in their study description.*** *Are gender, human rights and socio-economic equity perspectives adequately taken into account?* ***Not discussed.*** *Is research capacity strengthening addressed accurately?* ***Not discussed.***

Gender equity aspects in relation to:

a) Study participants: the study has been designed to ensure an analogous participation of men and women across the various sub-populations considered in this study: Men and women from the general population; MSM, transwomen, male and female sex workers, people living with HIV.

b) Research questions: the project aims to improve HIV and SRH in a framework of integration which follows principles of human rights and gender equity.

c) The designated research team: our research team is gender balanced.

Community participation

The research projects, from its inception includes the different study communities. It will engage with clients in focus group discussions and perform key informant interviews with key stakeholders from each study community in the formative phase of the project, so that the intervention is adapted to the local context.

Research capacity strengthening

Besides strengthening the country team's research capacity in qualitative methods and brief intervention training, the project will involve health professionals from the study sites in a variety of qualitative research methods such as focus group discussion, in depth interviews, theatre testing and cognitive interviews, enabling high quality formative research for future projects and qualitative research, as well as refresh quantitative research methods, as part of the institution's effort to develop research capacity in the public sector.

CV, TIMELINE AND BUDGET: *Do the CVs of the investigators match project needs?* ***Yes*** *Is the project timetable comprehensive and realistic,* ***Need a few more months – see comments above*** *are the proposed costs feasible and relevant to the scope of the study?* ***Need budget for a few more months – see my comments above.*** *Well justified?* ***Yes***

OVERALL ASSESSMENT AND FEASIBILITY: *Please comment on the overall quality of this project.*

Important, well written project. Maybe a bit too ambitious taken the time period into account. Qualitative research takes time!

RECOMMENDATION (CHOOSE FROM BELOW): 2

1. Approval
2. Contingent Approval-revisions or amendments required or subject to clarification
3. Rejection

IF YOUR RECOMMENDATION IS A 2 OR 3 PLEASE PROVIDE ADDITIONAL COMMENTS IN THE SPACE BELOW:

1. The protocol I have received seems not to be the final one since the track change mood is still on in some parts of the protocol. Also in some parts it seems the text is just cut and pasted from the generic protocol and not totally related to the actual study. One example on page 27 is "During Phase I, approximately 45 IDIs with providers will be conducted in each country by members of the in-country study team. IDIs will be stratified by study site and provider type, with a total of 15 IDIs for each study site (see Table 1)." This information is not available in table 1 but I think it should be. Further, in the heading of table 1, 2, 3, 4 and 5 you should write that the numbers are for your study in Peru and not "in any country". The referral to table 3 on page 30 should actually be to table 4.
   - The track changes were there to show how the site-specific protocol differed from the core protocol (that already has RP2 and ERC approval). Thank you for these suggestions, amendments have been made.

2. The context of the brief intervention presented in fig 1: The time allocated to each step in the counselling session seems very detailed. All clients might not need same number of minutes for each "theme". Counselling is a process where the counsellor does not tell the client what to do but more of a discussion as you point out in the introduction to the study. I doubt that the counsellor can follow this detailed plan fully. What about individual needs? What will happen if a client need more "minutes" and e.g. ask questions? Although this is a feasibility study that will show possible shortcomings of the intervention, I strongly suggest that you think through how realistic this detailed time plan is and plan for how to support individual needs recognized during the counselling session. I also wonder if you will be able to reach your goal in only one session? If needed, can you add counselling during the two follow-up surveys?
   - We agree with this point – the times are averages based on our previous experiences with brief interventions. We acknowledge that clients will progress through the intervention at different rates and will experience each stage of the intervention differently.
   - The intervention is client led – if the client requires more time on one stage/ one set of questions, then this will be allowed: the counsellor will not force them on to the next set of questions. The outline is a FRAME for the counselor to follow – with training the counselors will be able to move the clients through the session, while being led by the needs of the client.
   - The detailed time plan is for training and counsellors will be trained to allow this to vary when implementing

3. During your previous assessment phase, you found that people had large and diverse needs not taken care of. Have you used this information and tailored your counselling intervention in relation to those needs? What about those whose needs differ from your detailed counselling intervention? Will they be referred somewhere else?
   - Yes, the intervention outline is a framework – the CONTENT of the questions and discussions to be had will vary by the needs of the client. This will be covered in the training, which includes detailed role playing across a range of client types and needs. The general intervention framework will be adapted to the needs of our specific local subpopulations.

4. The description of the study populations is not totally clear. Why are only women with unintended pregnancy included in the group “people living with HIV” on page 15? Later on in the protocol e.g. in phase II client cognitive interviews it seems “people living with HIV” are not included but “people seeking care for STIs”. Please clarify and use same wording everywhere in the protocol when you describe the different study populations. Why is no text presented under 2.3.6 and 2.3.7?
   - Thank you for these comments. During the adaptation, some inconsistencies got into the protocol. It has now been corrected everywhere.

5. According to the protocol, the 3-months follow-up surveys in phase IV after the intervention, which is stated to be a pilot study, will measure e.g. feasibility and sexual health outcomes. The samples of different client categories are decided from a qualitative perspective and you also state that you will not be able to detect powered changes in sexual outcome. Thus, I don´t see the relevance of including “sexual outcome” such as reduction of risky sexual behavior as an outcome. It might just be wasting time and money if you can´t draw conclusions. Why do you want to measure if feasibility changes over a 6-months period? Is that likely to happen? Do you emphasize any problems finding the same clients after 6 months?
   - We don’t expect to see statistical differences in our sexual health outcomes, but we need to measure these so that we know how to power the efficacy trial that is to follow. The primary outcomes are feasibility, the secondary outcomes are sexual health. The idea is not to draw final conclusions but to obtain an estimation of effects, if possible.
   - It is often not a problem to achieve changes in short periods of time – what is more challenging is to achieve intervention effects that are sustained. For that we need a longer intervention in a trial. Such larger, controlled trial may follow this pilot study, if appropriate.
   - We have considerable experience in cohort studies, with attrition kept generally below 20%.
6. I probably have a bit different definition of trustworthiness than what you have. Credibility might be another word to describe what I meant. “How well aim, data and analysis fit together. Relates to the quality of the research process relating to data collection and analysis. Triangulation i.e. using more than one method and having several persons analyzing data are ways to ensure credibility”. “How well others can follow what has been done during the process, e.g. how well everything is documented.” “Showing that the interpretation of data represents the reality of those studied. Quotes can be a way of showing this.” I think you are planning to do it in this way. Just good to think about these aspects when you collect data, analyze data and write it up.
   - We appreciate this comment and will explore these methods during implementation.

7. I might also have a different definition of inductive and deductive codes where my understanding is that deductive codes come from the actual themes in the FGD while inductive codes are more data driven, coming from the actual data (grounded in the data). These are then more intrinsic. The same aspect relates to analysis of exit interviews on page 53.
   - We take deductive from our theory and inductive from the data, as is the gold standard in qualitative research.

8. On page 6 you write about Survey Domain 3: Efficacy and Autonomy. But further down in the text you write Efficacy and Integration. Please clarify.

- This should be changed to integration throughout, thank you for noticing, it has been changed now.

9. Do you foresee any bias regarding the observations of the counselling sessions? Since there will be few observations the providers will not have time to get use to the observer but probably do their best to satisfy the observer. But this might be the goal for the observation??
   - We have used this technique in the past – we find that people can only change what they know to be wrong. For example, if they do not know it is wrong to use potentially derogatory terminology then they will not change it. We will report the limitation of this method when we report the results
   - This is a standard problem of the observation technique. It can be addressed by avoiding its use as a core evaluation technique, and, rather, emphasize its utility to assess the general approach of the provider, their basic capacity to address the topics of interest and interact with the client. Similarly, it may be very useful to identify issues in the counselling session that may be subsequently reinforced/resolved with the provider. In fact, the latter way of framing the observation will likely be more acceptable/less threatening to the provider.

10. In the Pre/Post Surveys with Clients, Survey Domain 6: Sexual Health Indicators the numbering is wrong. Further, as I mentioned earlier in point 5 I question the usefulness of this data. Sample size and sampling method do not support any conclusions here. You state that "Although this phase is not powered to detect statistically significant differences in the sexual health outcomes, this analysis will provide information on whether the intervention is associated with differences in these outcomes from baseline to three and six months' follow-up". To build future big RCTs on these rather shaky assumptions is according to my view not correct. Although it can be a way to create a hypothesis for a future full-scale intervention but that is all.
    - The numbering has been adapted
    - There is no baseline data for these populations in this context – so including sexual health outcomes in this study at least provides us with some data on which to build the hypothesis and inform the power for a larger study.
    - We thus agree with the reviewer in the potential utility of this pilot: it can confirm that there may be some sexual health outcomes, and may provide an indication of the effect size that can be used for hypothesis formulation and sample size calculation in a subsequent trial.
11. Regarding project time line, I suggest that you start analysis of data for phases I and II already during data collection, don´t wait until phase III. Since it is qualitative data you might want to change some things in the design and also use data from phases I and II in phase III. Furthermore, it seems that you have not included any time for writing up your report and preparing for dissemination. I think you need at least two more months in the end of the project to finalize analysis and write up everything. Then you also need to add budget for personnel cost during a couple of more months during year 2.
    - The time line has been adapted, thank you for the suggestion.
    - We added two more months at the end of the study, to complete analysis and ensure appropriate time for report preparation, and added some budget.

Second reviewer:

REVIEWER NAME: Anna Glasier

REVIEW DATE: 2017-11-20

REQUESTED BUDGET: US$ 167,000.

PROJECT DURATION (IN MONTHS):14 months DATE OF REVIEW: 20/11/2017

PROJECT SUMMARY: The core protocol for this study has already been approved. Site specific changes to the core protocol include only details of the populations to be recruited, the sites, the numbers of interviews/FGDs and the investigators. All the changes are acceptable.

Minor:

On page 10 section 1.6 The third bullet point appears to run out of steam ending with family planning, pre............

- Thank you for this comment, it has been amended.

On page 15 section 2.3.4 the word travesti is included. Is this a Peruvian term or do they means transvetites?

- Travesti is the Spanish-language term (in fact, originally from Italian) for cross-dressers and transvestites.

PROJECT SIGNIFICANCE: Already reviewed for the core protocol.

OVERALL PROJECT DESIGN: Already reviewed for the core protocol.

SAMPLING PROCEDURES: Already reviewed for the core protocol.

DATA COLLECTION: Already reviewed for the core protocol.

ANALYSES, MAIN VARIABLES or OUTCOMES: Already reviewed for the core protocol.

RESEARCH STANDARDS: No changes from the core protocol

CV, TIMELINE AND BUDGET:

The investigators seem well qualified and capable of doing the study.

The budget is presented with items identified but not justified with any accompanying text (which would have been helpful particularly with respect to the precise tasks for each of the personnel. However the budget seems to be reasonable. Just one quibble, office materials appears as a line item in Section 7 Other costs but printing and stationery supplies also appear in section 2 Services and small supplies. I feel this is duplication.

- Thank you. The item in section 2 is referred to printing of instruments for field work, while the item in section 7 is referred to materials for office use.

OVERALL ASSESSMENT AND FEASIBILITY: *Please comment on the overall quality of this project.*
