# Supplementary material for: Brief Intervention to Prevent Sexually Transmitted Infections and Unintended Pregnancies: Protocol of a Mixed Methods Feasibility Study
Source: JMIR Res Protoc. 2020 Mar 10;9(3):e15569. doi: 10.2196/15569 (PMC7093772; doi:10.2196/15569)
Supplement: Multimedia Appendix 1 [file resprot_v9i3e15569_app1.pdf]

FOR WHO USE ONLY

Date received by WHO:

Thematic area:

"Core" proposal (for multicentre study)

Connect ID No:

1. **Project title: Protocol for a Feasibility Study of a brief sexuality-related communication (BSC) intervention to Prevent STIs and Unintended Pregnancies**
   A-ID: Not given

Core protocol

REVIEWERS: Anna Glasier and Elisabeth Faxelid

REQUESTED BUDGET: Not given

RECOMMENDATION (CHOOSE FROM BELOW):

1. **Final Approval**

Dr Anna Ekéus Thorson MD, MPH, PhD, Prof **I Research Manager I Research Capacity Strengthening I HRP Alliance I** Department of Reproductive Health and Research I Office: +41227911393 Cell: +41792173401

RESPONSE TO REVIEWER'S COMMENTS:

Round two remaining comment by reviewer 2 (response approved by the research manager) :

1. I don´t understand how the authors define inductive and deductive codes and themes. According to my knowledge an inductive approach means that the codes and themes derive from the empirical data and a deductive approach that the analysis is directed according predefined themes. Is that what the authors refer to?

Inductive coding refers to the process in which codes to applied to the f qualitative data without any predetermined idea about which codes will be used in the process. Deductive coding is the process in which codes are pre-determined and applied to the qualitative data. In the proposed analysis of the qualitative data we will use both types of code: deductive codes will be drawn from the IMB theoretical framework, and will center on Information, Motivation and Behavior. Inductive codes will be those that arise organically from the data, and will pertain to cultural attitudes, norms and perceptions around the proposed intervention.

Comments by reviewers with point by point answers:

1. Justify the need for a full-blown feasibility study rather than an RCT with a preparation phase. We thank the reviewers for the opportunity to expand upon this point. While the effectiveness of brief interventions is well documented, little is known about how brief interventions should be tailored to different populations. We inserted the following on page 5 to expand upon this point:

However, there is little known about how brief interventions should be tailored for different populations. While the proposed brief intervention aims to be largely standardised across three populations at elevated risk for STIs and/or unintended pregnancies, the degree to which the intervention should be tailored to each population versus standardised to ease implementation is unknown. Secondly, the large-scale implementation of the proposed intervention would benefit from knowledge of how providers in the study communities view their ability to embed the intervention in their practice settings and health system environments. Characterised by competing demands for limited resources and a shortage of many types of primary health care providers, the health systems of many low- and middle-income countries may be initially ill-equipped to widely implement the proposed intervention- thus requiring alterations to the brief intervention prior to a multi-site randomised control trial of its effectiveness.

2. Clarify whether recruitment will be from five communities in each study centre (or country) or whether there will be a total of five study centres.

   Thank you for allowing us to clarify this. Recruitment will come from two to three communities in each study country. Each study country will select its study communities

according to national sexual health priorities within the bounds of the proposed client populations and sexual health outcomes. The number and names of the study countries have not yet been finalized by WHO. This is now included in section 2.4 on page 14.

The proposed feasibility study will take place in 2-3 communities per study country. The exact number and names of the study countries are not yet finalized. Each study country will select its study communities according to national sexual health priorities within the bounds of the proposed client populations and sexual health outcomes.

3. If there are five communities in each study site how many sites are proposed for this work?

   There are two to three communities in each study country. The number of countries has not yet been finalized by WHO, but we anticipate approximately 12 communities across all study countries.

4. Clarify whether the same providers and clients will be used for all four phases of the feasibility study.

   Thank you for allowing us to clarify this point. Providers and clients will not be precluded from participating in more than one phase of the feasibility study, but recruitment efforts will continue throughout all four phases. The following is now included in section 2.4 on page 14:

   Recruitment will occur with the assistance of key stakeholders and continue throughout all four phases of the feasibility study. That is, while participants from one phase will not be precluded from taking part in successive phases, recruitment of both clients and providers will be ongoing

5. Please provide a timeline or at least a sample timeline

   This is included as an annex in the latest submission

6. Please provide a ball park figure for a sample budget

   A sample budget is included in the latest submission. The sample budget is site-specific and includes the costs for the research activities only, and does not consider reimbursements for study participants. We estimate approximately US$35,000 per site for the duration of the study.

7. Please write the first section of the consent form in simple English.

   All consent forms have been simplified and the Flesch-Kinkaid reading level reduced. These are now included in the latest submission.

8. Please clarify whether the Intervention Manual as presented in Annex 2 is the manual which the primary care providers will receive and if it is re-write it in more appropriate language.

The manual presented in Annex 2 is the training manual, not the intervention manual. The attached therefore describes the intervention in detail and will be used by those training the providers on how to deliver the intervention. Providers themselves will receive a simpler set of materials that outline the intervention in a less technical manner.

REVIEWER TWO:

a. Clarify how the theoretical frameworks mentioned in the introduction will be used during analysis.
b.

The IMB model will be used throughout to guide the study, including in the analysis. Qualitative coding will use core IMB constructs to develop the preliminary codebook. Sections elaborating on the use of IMB in the analysis are now found on pages 29, 34, and 38.

Page 29: *A thematic analysis of the text will be completed, using elements of Grounded Theory [30] and building on the IMB model. This will include the systematic and consistent application of both deductive and inductive codes to the text. Inductive codes will include themes that are explicit domains present in the interview and FGD guides and deductive codes will include salient themes that arise more organically in the data. Additionally, all inductive themes and codes will be grouped according to the constructs in the IMB model, including information, motivation, and behaviour.*

Page 34: *After the CI process is complete, the in-country study team will synthesize the interviewers' notes according to the constructs of the IMB model to provide recommendations for changes to the BSC guide based on feedback given to interviewers during the cognitive interviews The research team will then make recommendations to the international study team on how the phrasing and language of the BSC guide should be altered to improve comprehension among providers and in all three client populations.*

Page 38: *All theatre testing discussions will be transcribed and de-identified. To analyse FGD data from Phase III, the same analysis process conducted in Phase I will be performed. This includes having multiple analysts apply preliminary codes to transcripts, until agreement is reached about the final codebook. Then, the final codebook will be applied to all transcripts, with at least two analysts applying codes to each transcript. Similar to Phase I, this codebook will include both inductive and deductive codes and will be grouped based on the IMB model.*

c. Write aim and specific objectives clearer in the beginning of Purpose, aim and objective section.

The objective of the study and aims of each phase have been revised to improve clarity.

Objective: *The objective of this study is to evaluate the feasibility of the BSC intervention to be implemented by providers working in primary health care centres with three specific client populations*

Phase I Aims (page 23):

- To *understand the feasibility of implementing the intervention among health care centres, providers, and clients*

- *To use in-depth interviews and focus group discussions to determine if content of the intervention should be altered to increase willingness, satisfaction, and acceptability of the intervention among providers and clients*

Phase II Aim (page 30):

- *To receive feedback from providers and clients regarding the language, phrasing, and delivery of the BSC Guide in order to improve acceptability of the study*

Phase III Aim (page 35)

- *To receive reactions from a convenience sample of clients and providers to the content, delivery, and materials used in the BSC Guide in order to obtain an assessment of acceptability and willingness regarding the feasibility study*

Phase IV Aims (page 39)

- *To measure provider knowledge, attitudes, and practice (KAP) regarding STI reduction and avoidance of unintended pregnancy in commercial sex workers, MSM, and individuals and couples attending clinics or organizations addressing STIs and unintended pregnancy*
- *Test the feasibility of the intervention, including provider and client willingness, satisfaction, and acceptability of the intervention and the research study*
- *Determine if the intervention led to increased self-efficacy, behaviour change, and reduced risk of unintended pregnancy and STIs among clients*

d. Elaborate more on the different methods to be used and the data collection process in phase 1-3, (especially methods in phase 2 and 3) – see my previous comments.

e.

The methods for Phases II and III are now more clearly written and expanded upon.

f. Clarify how and when the outcome "satisfaction with the intervention" is measured – see my previous comments.

The reviewer makes an excellent point. The outcome of satisfaction is now revised to be clearer in terms of its use in Phases I-III. Satisfaction in this sense refers to the perceived helpfulness, value, and utility of the intervention should the proposed changes be implemented. The following is now written on page 20:

Satisfaction is assessed at each phase of the feasibility trial, both in the formative qualitative work and the quantitative phase. .During the first three phases, satisfaction refers to the perceived helpfulness, value, and utility of the intervention should the proposed changes be implemented. In Phase IV, satisfaction refers to providers' and clients' satisfaction with the intervention itself.

g. Clarify analysis for phase 3 – see my previous comment.

The analysis for Phase III now includes specifics regarding the use of the theoretical model as well as regarding the specific qualitative approaches that will be used.

Page 39: *All theatre testing discussions will be transcribed and de-identified. To analyse FGD data from Phase III, the same analysis process conducted in Phase I will be performed. This includes having multiple analysts apply preliminary codes to transcripts, until agreement is reached about the final codebook. Then, the final codebook will be applied to all transcripts, with at least two analysts applying codes to each transcript. Similar to Phase I, this codebook will include both inductive and deductive codes and will be grouped based on the IMB model. The analysts will then create descriptions for each theme, identifying common concepts, patterns, and statements that appear throughout the discussion. Specific themes regarding content and delivery of the intervention will be used by the research team to further refine the BSC Guide, determine the best way to present content and better understand provider/client attitudes and willingness to participate in the BSC intervention. While small changes to the BSC Guide will be site-specific in order to promote cultural sensitivity, larger changes will be implemented universally across study sites.*

h. Include a short piece on how to deal with trustworthiness.

The reviewer makes an excellent point regarding the need for this section. This is now included on page 29.

Multiple measures will be taken to ensure that the qualitative data are trustworthy. All qualitative data collection activities will be reviewed by local advisors in order to ensure that they are appropriate for local contexts. This will help to ensure that participants feel safe in the interviews and FGDs and to ensure that the activities are all culturally appropriate. All interviews and FGDs will be conducted by a well-trained team, who will have skills in building rapport and creating a safe environment. During the consent process, confidentiality will be stressed to ensure that participants are aware that the data will only be used for the purpose of the research and will not be shared with anyone else. In addition, the guides follow a process where more general, less intrusive questions are asked in the beginning, so as to help build rapport before asking more difficult interview and focus group questions. Active listening skills will also be employed to help participants feel more comfortable. Taken together, these mechanisms should assist with increasing the trustworthiness of the qualitative data.

i. Include relevant informed consent forms – see my previous comments.

Additional consent forms were created and are included in this latest submission.

j. Clarify if ethical clearance is needed and in that case from where and for which parts of the project?

The proposed study will be approved both by the ERC of the WHO and the relevant ethics review boards of each study country once they are finalized. This is now included in the ethics section on page 18:

The protocol for the proposed feasibility study will be subject to approval by the Ethics Review Committee (ERC) of the World Health Organization, as well as the appropriate ethics review boards of each study country.

DATE OF REVIEW: 13.04.2017

PROJECT SUMMARY: This proposal is for a multi-phase, multi-site feasibility study as a precursor to a larger RCT that aims to change the sexual risk behaviour of three vulnerable populations (commercial sex workers, men who have sex with men and (mainly young, unmarried) heterosexual individuals and couples). An intervention that is largely standardized for all three population groups, tailored to the specific sexual health issues facing each key population (STIs and unintended pregnancy) is proposed.

The study comprises three phases

1) A Brief Sexuality-Related Communication (BSC) has already been developed. It consists of an overview of the technical principles of Motivational Interviewing (MI) and its application to BSC. It then delineates the intervention into eight components collectively lasting approximately 25 minutes.
2) A **feasibility study -** involving formative research to understand the local contexts of each study setting, and evaluate the willingness, acceptability, safety, and logistical barriers/facilitators of delivering the BSC intervention to healthcare providers and clients. The BSC guide will be revised and refined throughout the phases of the feasibility study.
3) Multisite randomized controlled trials – to evaluate the effectiveness of adapted versions of the BSC intervention to prevent STIs, including HIV, and unintended pregnancy in primary healthcare settings.

This proposal presents the feasibility study which comprises four phases and will be jointly implemented by an international team of researchers as well as in-country study staff who have specific knowledge of the study populations and healthcare system in each study community. The objective of the feasibility study is to evaluate the feasibility and acceptability, both to healthcare providers and clients (from the three populations groups), of the BSC intervention to prevent STIs including HIV and unintended pregnancies.
The feasibility study comprises four phases:

- **Phase I** : Focus Group Discussions (FGD) with clients representing each key population of interest from each study community, in-depth interviews (IDI) with providers at each site clinic, and key informant interviews (KII) with stakeholders in each study community to determine willingness, acceptability, and satisfaction with the intervention will be asked.
- **Phase II** uses cognitive interviewing (CI), in which feedback is sought regarding the language, phrasing, and delivery of the BSC guide.
- **Phase III**, theatre testing, provides a mock BSC session demonstrated by in-country study staff. The aim of this phase is to obtain an assessment of willingness and acceptability to participate in the feasibility study from a representative sample of the three study populations. The participants will provide feedback on the content, delivery, and materials used in the BSC intervention and engage in group dialogue surrounding potential improvements to the guide. **Providers will attend a four day training after Phase III and before starting Phase IV**
- **Phase IV** is a non-randomized pre-posttest trial of the BSC intervention in which clients and providers will be followed for 6 months with follow-up surveys every three months to measure the same constructs as Phases I and III as well as secondary sexual health outcomes.

The introduction to the proposal finishes saying that MI-delivered interventions to prevent HIV, STIs, and unintended pregnancies are effective among different populations and in different socio-cultural contexts. It adds that WHO has conducted a systematic review of the literature and that brief MI-delivered sexual risk reduction interventions (5-40 minutes) were effective when delivered in a single session format or in a 1 session + 1 boost session format

- These brief MI-delivered interventions were acceptable and feasible at primary healthcare level to both providers and clients and can be adapted to different socio-cultural context
- MI-delivered interventions are effective when targeting different populations such as adolescents, gay and bisexual men and people living with HIV
- MI technical principles can be used, and were effective, in preventing HIV/STIs when the brief intervention was delivered using multimedia technologies.

If this is the case, than why is it necessary to conduct a feasibility study? Why not simply go straight to the RCT accepting that this type of intervention has been shown to be effective? We are not told what the budget for this study will be and nor can I find a timeline (including in the full protocol annex) but it is a complex feasibility study and will surely be costly. For me the authors need to justify the need for doing

a feasibility study at all when these types intervention has been shown to be acceptable, feasible and effective

PROJECT SIGNIFICANCE:

The significance of the project is very well described. Arguably the authors use valuable space in describing the significance in detail which could have been used to provide more details of the study itself. The research clearly links to research agendas of relevance to SRHR.

Will it provide new knowledge? - This is a feasibility study, MI has already been shown to be feasible. I suppose that new knowledge will be provided but one has to ask whether this knowledge is strictly speaking necessary.

OVERALL PROJECT DESIGN:

The project is well described although some vital information is missing from the project description for RP2 and I had to go to the annex containing the full proposal to find the information e.g. sample sizes for each of the participant groups.

SAMPLING PROCEDURES: Time is spent defining MSM, CSW and the third group of interest together with key informants, but there is no mention of sample size for any phase of the feasibility study in the document intended for RP2 review.

DATA COLLECTION:

It is not clear to me whether the same set of providers and/or clients will be recruited for all four phases of the study. The full protocol lists five communities where the research will take place. I am not clear whether this will be five communities in each centre/country or five centres (countries) in total. If it is the former then it may be difficult to recruit sufficient providers particularly as this research is quite demanding of their time before Phase IV even starts. As someone who ran a large SRH service I would have found it very difficult to release staff for this size of project without jeopardising the delivery of the routine service. Similarly the demands on the clients are quite steep.

This is a complicated study, it will take a lot of organisation and oversight.

ANALYSES, MAIN VARIABLES or OUTCOMES:

This section is very well written and presented (although again I had to go to the full protocol for some of the vital information. The tables are particularly well done and helpful

RESEARCH STANDARDS:

I do not see any ethical challenges. The consent form contains some pretty sophisticated language e.g. the phrases in colour in the following paragraph. 'You are being asked to participate in a discussion today that will help researchers at the World Health Organization better understand your perceptions of an intervention designed to reduce sexually transmitted infections (STIs) and unintended pregnancies in your community. The study consists of a 25-minute counselling session with your primary health care provider or pharmacist that is designed to help people set and

achieve specific goals for engaging in safer sexual behaviours and avoiding STIs and unintended pregnancy.

While of course consent forms will be translated into local languages unless they are simple and clear in English at the outset then there is a danger that a verbatim translation will be even less clear (and we will never know because we do not speak the local language)

Research capacity strengthening is not addressed.

CV, TIMELINE AND BUDGET:

This is a core protocol so there are no details of investigators. I assume that once sites are identified this aspect of the proposal will need to be reviewed together with the budget. There is no timeline for the proposal that I can find. While it is not a problem that there are no details of researchers (nor even proposed countries) I do think we need to see a timeline and a ball park figure for a budget.

OVERALL ASSESSMENT AND FEASIBILITY: This is a well written proposal but it is complicated and demanding. Since no centres have yet been identified it is going to be hard for the researchers to know whether the study is manageable.

My main concern is that there is no justification for doing a feasibility study when MI has already been shown to be feasible and effective. I would prefer to see a proposal for an RCT which includes a preparatory phase (including training in MI and the intervention).

I find the Intervention Manual (Annex 2) almost impenetrable. I find it hard to imagine that a primary care provider (nurse, pharmacist or lay counsellor) will even start to make sense of it. Perhaps I have misunderstood the audience for Annex 2. I realise that feedback on the BSC guide will be collected in Phase II of the intervention study but you could save a lot of time by making this a user-friendly manual before presenting it to the providers for their first review.

RECOMMENDATION:

2. Contingent Approval-revisions or amendments required or subject to clarification

IF YOUR RECOMMENDATION IS A 2 OR 3 PLEASE SPECIFY IN THE SPACE BELOW:

9. Justify the need for a full-blown feasibility study rather than an RCT with a preparation phase.

10. Clarify whether recruitment will be from five communities in each study centre (or country) or whether there will be a total of five study centres.
11. If there are five communities in each study site how many sites are proposed for this work?
12. Clarify whether the same providers and clients will be used for all four phases of the feasibility study.
13. Please provide a timeline or at least a sample timeline
14. Please provide a ball park figure for a sample budget
15. Please write the first section of the consent form in simple English.
16. Please clarify whether the Intervention Manual as presented in Annex 2 is the manual which the primary care providers will receive and if it is re-write it in more appropriate language.

1. **Project title** **Feasibility Study of a Brief Intervention to Prevent STIs and Unintended Pregnancies**

   A-ID:

2. **Principal investigator NA**

REVIEWER NAME: Elisabeth Faxelid

REVIEWER NAME:

REQUESTED BUDGET: Not stated

PROJECT DURATION (IN MONTHS):

DATE OF REVIEW: 21st April 2017

INSTRUCTIONS: *Please review the proposals on the following criteria, focusing on major strengths and weaknesses. Comments should be typed in the spaces below each section. Type as much as needed, the document will allow for unlimited text.*

PROJECT SUMMARY: *Please provide a brief statement of the main aim and objectives. Is there a good review of the literature, including reference to a systematic review if applicable, justifying the research questions?*

This proposal starts with a rather long and comprehensive text related to purpose, aim and objectives with the following research question: "Is a standardized Brief Sexuality-Related Communication (BSC) intervention feasible and acceptable to healthcare providers and all three key populations? The main objective is to evaluate the feasibility and acceptability, both to healthcare providers and clients, of the BSC intervention to prevent STIs including HIV and unintended pregnancies. The project starts with a qualitative part involving three types of clients, providers from different settings and different types of community stakeholders. The whole project is guided by the Information-Motivation-Behavioural Skills Model, which includes aspects from the Theory of Reasoned Action and from the concept self-efficacy. Aim and objectives could have been stated clearer here. There is, however, a table presented later on in the proposal, which includes specific objectives related to the different parts of the project. A literature survey of the filed follows with relevant references included to justify the stated research problem under study.

PROJECT SIGNIFICANCE: *Is the significance of the project accurately described? Does it include links to research agendas of relevance to SRHR? Will it provide new knowledge?*

The significance is well described and the research is relevant to SRHR. Hopefully new knowledge will follow from intervention results.

OVERALL PROJECT DESIGN: *Is the proposal scientifically sound and is the design being proposed the most appropriate to accomplish the stated objectives? Please provide a critique of the conceptual approach and the proposed project structure in relation to the objectives the project seeks to achieve. Have checklists (STROBE, PRISMA, etc) accurately informed the methods description?*

The first 3 phases are mainly qualitative. I have concentrated on these phases. Methods to be used in phase 1 are focus group discussions (FGDs), in-depth interviews with providers and key informant interviews with community stakeholders. These methods are relevant. But they are described briefly and in a very general way. Three groups of clients will be included (commercial sex workers, men who have sex with men and individuals/couples who seek care for STI or unintended pregnancy) in the FGDs. The three groups are defined but how they will be selected is presented only in a general way. Will these groups form different FGDs? How many FGDs? Probably not wise to have couples in same FGD. Different types of providers in primary health care settings will be selected for in-depth interviews. Selection/sampling is described in a general way. The relevance is thus not possible to judge for individual contexts. Number of persons to be interviewed is not presented. Key informant interviews will be carried out with different types of community stakeholders. Selection/sampling is described but in a general way. The relevance is thus not possible to judge for individual contexts. How to decide the number of person that need to be interviewed is not presented. Why are providers interviewed in-depth and stakeholders and clients not? How will the in-depth interviews and key informant interviews differ? All groups are going to be asked about willingness, acceptability and satisfaction with the intervention. The results regarding these concepts will be the outcome(s). Phase 1-3 is carried out before the actual intervention and it is thus not clear how “satisfaction with the intervention” can be an outcome. Make

this clear in the text. The table “data collection methods” gives a good overview of study population, methods and aims. The text describing all parts of the project could have been written according to the table content to make the design including methods clearer. Now the information comes in different places and thus a bit difficult to follow.

In phase 2 “Cognitive interviews” will be used and “theatre testing” will be used in phase 3. These methods are not clearly described. How does a “cognitive interview” differ from an “ordinary interview”? The method “theatre testing” using FGDs in phase 3 should also be described clearer. Again, recruitment and sampling of participants is described only in a general way (three study populations and providers in phase 2 and providers or clients in phase 3). It is thus not possible to judge the relevance. It is stated that in phase 3 study participants should be recruited in order “to get a representative sample of the three study populations”. Also in the text under the heading “ethical considerations and core values” the importance of representativeness is mentioned. Since phase 1- 3 are qualitative parts it is not totally appropriate to plan for representative samples. Representative samples are usually the characteristics of quantitative, not qualitative studies. This needs to be clarified.

SAMPLING PROCEDURES: *Is the sampling, (including representativeness, if required) and the sample size, adequately described and justified? Are adequate procedures outlined to recruit the respondents? If a qualitative study, is selection of participants and method for gathering data accurately described?*

Study settings are described broadly and in a general way. Although these settings are relevant, I would have liked to see the context in order to make a proper judgment of its relevance. Recruitment of participants is described in a general way. Sample size for phase 1-3 is not mentioned. See also my comments in previous section.

DATA COLLECTION: *Do you foresee any difficulties in the proposed field activities, especially in the plans for data collection?*

This is difficult to judge since the proposal is generic. There might of course be a number of challenges during data collection depending on the specific context. The proposed project organization should, however, be able to deal with specific problems that occur during data collection. Then it depends on how much time the different actors can allocate to the project implementation activities.

ANALYSES, MAIN VARIABLES or OUTCOMES: *Is information provided on methods of analyses? For quantitative studies, intended statistical methods? Are the main variables or outcomes properly described?*

I have read only the qualitative parts but I consider, however, that the different outcome(s) are well described.

For qualitative studies, are scientifically sound methods of data collection analyses described, aiming for robustness and trustworthiness.

Methods for analysis of the qualitative data, especially for phase 1 data, are well described. It is a bit unclear how the data from phase 2 and 3 (especially phase 3) will be analyzed. The authors state that the same codebook will be used for analysis of data in phase 1 and 3. Since the aims differ for phase 1 and 3 I doubt that then results will so similar that the same codebook can be used. Trustworthiness is

not specifically mentioned. A short description relating to what trustworthiness is and how it can be achieved should be added.

RESEARCH STANDARDS: *Do you see any ethical challenges in the project description? Are gender and socio-economic equity perspectives adequately taken into account? Is research capacity strengthening addressed accurately?*

Ethical considerations are described. How and if ethical clearance is needed for the different phases is not described. Two informed consent forms are included in annex 3. The first one relates to the FGDs. The form includes everything but there are repetitions in the text and the part "study overview" is a bit confusing since it mentions the phase 4 study. I suggest that this informed consent focus on the FGDs and also that it is structured so text it not repeated. The second informed consent form relates to evaluation of phase 4 and a survey. Make it clear for the participants that they will be interviewed and for how long and by whom. No informed consent document is presented for providers and stakeholders. Is that not needed? No informed consent is presented for data collection in phase 2 and 3. Informed consent forms for clients included in those two phases should also be prepared.

Forming FGDs with clients having STIs and/or unintended pregnancy might be an ethical challenge. It is stated that a mix of gender and social profiles will be envisioned. It is, however, not mentioned if men and women and persons with different socio-economic status will be in same FGDs or not.

CV, TIMELINE AND BUDGET: *Do the CVs of the investigators match project needs? Is the project timetable comprehensive and realistic, are the proposed costs justified?*

The timetable seems realistic. Budget/costs is not included although budget posts are presented. No specific CVs but a generic CV is presented. A suggested project organization is also presented. It is, however, not possible top judge the relevance of these aspects.

OVERALL ASSESSMENT AND FEASIBILITY: *Please comment on the overall quality of this project.*

The current proposal is written in a very general way and it is very broad. Although the whole idea behind the proposal is fine, the proposal is more like a cookbook and as such it is difficult to judge feasibility without knowing the specific study settings. The qualitative parts (phase 1-3) need some adjustments and clarifications. See my comments under overall project design, sampling and analysis.

RECOMMENDATION (CHOOSE FROM BELOW): 2

1. Approval
2. Contingent Approval-revisions or amendments required or subject to clarification
3. Rejection

IF YOUR RECOMMENDATION IS A 2 OR 3 PLEASE SPECIFY IN THE SPACE BELOW:

k. Clarify how the theoretical frameworks mentioned in the introduction will be used during analysis.
l. Write aim and specific objectives clearer in the beginning of Purpose, aim and objective section.
m. Elaborate more on the different methods to be uysed and the data collection process in phase 1-3, (especially methods in phase 2 and 3) – see my previous comments.

n. Clarify how and when the outcome “satisfaction with the intervention” is measured – see my previous comments.
o. Clarify analysis for phase 3 – see my previous comment.
p. Include a short piece on how to deal with trustworthiness.
q. Include relevant informed consent forms – see my previous comments.
r. Clarify if ethical clearance is needed and in that case from where and for which parts of the project?
