# Supplementary material for: Brief Intervention to Prevent Sexually Transmitted Infections and Unintended Pregnancies: Protocol of a Mixed Methods Feasibility Study
Source: JMIR Res Protoc. 2020 Mar 10;9(3):e15569. doi: 10.2196/15569 (PMC7093772; doi:10.2196/15569)
Supplement: Multimedia Appendix 3 [file resprot_v9i3e15569_app3.pdf]

20, AVENUE APPIA – CH-1211 GENEVA 27 – SWITZERLAND – HTTP://WWW.WHO.INT/ETHICS/REVIEW-COMMITTEE/EN/ – HTTPS://EXTRANET.WHO.INT/ERCWEB/LOGIN.PHP

WHO ERC
Review Summary

Protocol ID: BSC.1
Country: Master protocol
Protocol Title: Feasibility Study of a Brief Intervention to Prevent STIs and Unintended Pregnancies
Version: 2 **Dated:** 07/05/2017
WHO Responsible Staff Member: Karel BLONDEEL
Responsible Unit: WHO/HQ/FWC/RHR
Meeting Date: 03/08/2017

Dear Dr. Karel BLONDEEL,
Please find the review summary of the Protocol "Feasibility Study of a Brief Intervention to Prevent STIs and Unintended Pregnancies", which was submitted to the Secretariat on 26/05/2017. This proposal underwent regular review.

The outcome of the review is provided below. When responding, please submit the following:

1. A cover memorandum that addresses your responses, POINT BY POINT, to each of the queries in sections A and B.
Section C contains Suggestions to improve the proposal but there is no obligation to follow them.

2. An Amended protocol including the responses in bold, highlighted or in track changes. Please ensure that tracking formatting changes is switched off or that all formatting changes have been accepted and that no comments which the team may exchange during the editing are included in the track changes version. The protocol should include all relevant documentation (ICF, study instruments, peer review, etc.) even if already submitted.

Please note that comments in the introductory paragraph are meant for the WHO Responsible Staff Member, though you may decide to share them with the PI.

PLEASE RESPOND TO THIS REVIEW SUMMARY WITHIN A three MONTH PERIOD, OR PROVIDE THE ERC SECRETARIAT A VALID JUSTIFICATION FOR THE DELAY.

The ERC understand that this is a Master Protocol and that site specific proposals will follow. However, further information is required in terms of identification of potential participants, recruitment and consent process, etc. Please find specific comments below.

A. Amendments (Response and change required)

This section includes queries and comments on your protocol, study instruments or the informed consent form for which the ERC requires your response and where relevant, appropriate amendments to the protocol, study instruments or the informed consent.

1. Protocol

1.1. Please provide an amended proposal specifying the version number and/or date on each page.

1.2. One of the study populations is "individuals or couples seeking care for STIs and/or unintended pregnancy". People seeking care for STIs and those who have unintended pregnancies may be quite different populations with different basis for their attendance and different health seeking behaviour . The first group may consult an STI clinic for example and the second an antenatal clinic. Please review if it is appropriate to group them and modify as necessary.

1.3. Section 1.6 states "The majority of unintended pregnancies in low and middle-income countries occur among women ages 15-24, many of whom also present with sexual risk behaviours that elevate their risk of STIs and HIV". Given that this is a high risk population that could potentially benefit the most from the intervention, it is unclear why the age of inclusion criteria is 18-45 years. Excluding adolescent, high risk

women seems inappropriate and an important omission. The ERC encourages the study team to include minors. Please address.

1.4. Please specify what will be the compensation offered to participants to the study and advise whether such compensation is likely to be a motivating factor for recruitment.

1.5. Venue-Based Sampling" is proposed to select participants. By "Using a pre-determined protocol, study staff will approach every fourth person who crosses a specific perimeter and assess their eligibility to participate and interest in joining the study." This seems a difficult approach especially outside of clinics that risks stigmatisation. Please clearly describe how this will be done while preserving privacy, confidentiality, and safety of researchers and participants.

Please note that when submitting site specific protocols, the information listed below should be clearly specified:

1.6. The linkages that were made with different partners to conduct the study (i.e. research institutions, MOH, etc.) as well as roles and responsibilities of each one of them.

1.7. Who the stakeholders assisting in client recruitment will be.

1.8. Given the degree of discrimination and stigma related to some of the study populations will vary from one country to another, please explain how identification of potential participants will be done while preserving privacy and confidentiality. This is particularly important given that the study proposes to include different groups of participants from the same institutions.

1.9. The consent process that will be followed with the different groups (i.e. who will consent participants, when, where, etc.).

1.10. The compensation that will be offered to participants.

2. Study Instruments

2.1. It is noted that some study instruments are under development (i.e. intervention manual). Please submit when available.

3. Informed Consent Forms

3.1. Under "Study overview"

3.1.1. Please provide further description / information related to the study.

3.1.2. Rather than stating the objective of the study as to "design an intervention to reduce sexually transmitted infections (STIs) and unintended pregnancies" it could be conveyed as "to promote clients' sexual and reproductive health by improving communication between clients and providers" (as stated under "Benefits").

3.2. Under "Procedure" please clarify what is meant by "intervention".

B. Clarifications (Response required but change may not be required)

This section includes queries on your protocol, study instruments or the informed consent form for which the ERC requires a clarification, and it may not be mandatory for you to make changes to your protocol. Please consider the comments of the ERC and determine if you believe change is needed. If no change is made, the ERC will consider the response. If the judgement of the ERC is that a change should occur, the ERC will promptly notify you.

1.1. The protocol states that this feasibility study will take place in 2-3 communities per study country. Please specify the criteria that will be used to select sites, the target populations, and communities.

C. Suggestions

This section consists of suggestions for alternative scientific or technical approaches or methods for conducting the research but which do not raise critical, ethical issues. These are meant to be helpful to investigators and are presented as suggestions for you to consider incorporating into a revised protocol. No response from you is

required for any comment in this section. If, however, you do make changes to the protocol as a result of these suggestions, please submit the revised protocol to the ERC.

1. It would be useful to add a list of acronyms and a glossary. Some words are used interchangeably (i.e. key informants versus key stakeholders).
2. It would be useful to add a paragraph describing what is understood by "cognitive interviews".

Information Sheet for Focus Group Discussion

3. Under "Voluntary participation and withdrawal from the study" suggest to rephrase "Your behaviour becomes destructive to the group discussion" by something similar to "Your participation may hinder the effective running of the discussion".
4. Under "Participants" suggest to re-structure such as: "You are being asked to volunteer for this focus group because you are between 18-45 years of age and have said that you are either:
   - a man who has sex with other men.
   - a woman who has traded goods or money for sex
   - a person seeking care for STIs at this healthcare centre, or
   - an individual/couple seeking care for unintended pregnancy at this healthcare centre.

20, AVENUE APPIA – CH-1211 GENEVA 27 – SWITZERLAND – HTTP://WWW.WHO.INT/ETHICS/REVIEW-COMMITTEE/EN/ – HTTPS://EXTRANET.WHO.INT/ERCWEB/LOGIN.PHP

Protocol ID: BSC.1 **Meeting Date**: 03/08/2017

Based on the above comments, the Committee has the following recommendation(s) for this proposal:

[ ] The proposal is ***Approved as submitted.*** No modifications are required.

[X] The proposal is ***Conditionally Approved; requires amendments and/or clarifications.*** Final approval is contingent upon an adequate response by the Principal Investigator, to the satisfaction of the reviewers or the Chair on behalf of the ERC.

[ ] The proposal is ***Not approved; requires additional information and/or rewriting.*** A revised version of the proposal should be re-submitted by the WHO responsible staff member as a new submission to the ERC for re-review by Committee.

[ ] The proposal is ***Rejected***. The proposal is ethically unacceptable, for the reasons stated above. The Principal Investigator may submit a new proposal that takes into consideration the ethical issues raised by the Committee. If you do not agree with the Committee's assessment, please feel free to submit an appeal to the Chair of the ERC, through the Secretariat.

NOTE: Final Approval of the Proposal is contingent upon submission of the following:

[ ] Local ethics approval(s) [ ] Other relevant documents

The ERC would like to receive a copy of the recommendations of the local ethics committee when available.

IMPORTANT

1. *Any changes to the proposal or to the attachments (informed consent/study instruments etc.) should be approved by ERC before being implemented.*
2. *The approval for this proposal is valid for a period of **one year only.***
3. *Please resubmit this proposal for a Continuing Review at least 2 months before the next re-approval period.*

Chairperson .... **Date... 09 August 2017 ...**

Name: Nigel Rollins

**FINAL APPROVAL**	**FOR THE SECRETARIAT**
Amendments and Clarifications to the proposal have been reviewed. The protocol (Version: 3 Date: 02.10.17) and informed consent Forms (Dated: 27.10.2017) submitted on ..06.11.2017........... are approved by the ERC<br><br>**Chairperson** ....<br>NR/KF<br>**Name** ....<br>**Date**	Amendments and Clarifications to be reviewed:<br>[ ] Electronically by ERC<br>[ ] by Primary reviewers<br>[ ] by Secretariat<br><br>Amendments approved / Clarifications accepted on<br>Local ERC approval(s) obtained on NA<br>Relevant Documents submitted on NA<br>Comments: NA<br><br>Signature Date 06.11.2017
